# A Systems Biology Approach To Disentangle the Direct and Indirect Effects of Global Transcription Factors on Gene Expression in Escherichia coli

**DOI:** 10.1128/spectrum.02101-22

**Published:** 2023-02-07

**Authors:** Mahesh S. Iyer, Ankita Pal, K. V. Venkatesh

**Affiliations:** a Department of Chemical Engineering, Indian Institute of Technology Bombay, Mumbai, India; University of Minnesota Twin Cities

**Keywords:** global transcriptional factors, transcriptional regulatory network, regulatory cross talk, TF-TF epistatic interactions, metabolite-TF interaction, pleiotropy

## Abstract

Delineating the pleiotropic effects of global transcriptional factors (TFs) is critical for understanding the system-wide regulatory response in a particular environment. Currently, with the availability of genome-wide TF binding and gene expression data for Escherichia coli, several gene targets can be assigned to the global TFs, albeit inconsistently. Here, using a systematic integrated approach with emphasis on metabolism, we characterized and quantified the direct effects as well as the growth rate-mediated indirect effects of global TFs using deletion mutants of FNR, ArcA, and IHF regulators (focal TFs) under glucose fermentative conditions. This categorization enabled us to disentangle the dense connections seen within the transcriptional regulatory network (TRN) and determine the exact nature of focal TF-driven epistatic interactions with other global and pathway-specific local regulators (iTFs). We extended our analysis to combinatorial deletions of these focal TFs to determine their cross talk effects as well as conserved patterns of regulatory interactions. Moreover, we predicted with high confidence several novel metabolite-iTF interactions using inferred iTF activity changes arising from the allosteric effects of the intracellular metabolites perturbed as a result of the absence of focal TFs. Further, using compendium level computational analyses, we revealed not only the coexpressed genes regulated by these focal TFs but also the coordination of the direct and indirect target expression in the context of the economy of intracellular metabolites. Overall, this study leverages the fundamentals of TF-driven regulation, which could serve as a better template for deciphering mechanisms underlying complex phenotypes.

**IMPORTANCE** Understanding the pleiotropic effects of global TFs on gene expression and their relevance underlying a specific response in a particular environment has been challenging. Here, we distinguish the TF-driven direct effects and growth rate-mediated indirect effects on gene expression using single- and double-deletion mutants of FNR, ArcA, and IHF regulators under anaerobic glucose fermentation. Such dissection assists us in unraveling the precise nature of interactions existing between the focal TF(s) and several other TFs, including those altered by allosteric effects of intracellular metabolites. We were able to recapitulate the previously known metabolite-TF interactions and predict novel interactions with high confidence. Furthermore, we determined that the direct and indirect gene expression have a strong connection with each other when analyzed using the coexpressed- or coregulated-gene approach. Deciphering such regulatory patterns explicitly from the metabolism point of view would be valuable in understanding other unpredicted complex regulation existing in nature.

## INTRODUCTION

Global transcriptional factors (TFs) are the cornerstone in formulating an appropriate response to any environmental cue (1–4). While the importance of global TFs is well appreciated, the true direct targets and indirect effects on gene expression together with their role in particular environment remain unclear. The direct targets represent genes whose promoters have TF binding sites and respond consecutively to that specific TF gene expression or activity under a particular condition. In contrast, the indirect targets are genes whose expression is not directly influenced by the specific TF but which respond to the changes in TF in an unknown manner. Seminal studies have shown that these genome-wide indirect effects are primarily mediated by alterations in growth rates or the physiological state of the organism that encompass regulation by RNA polymerase-associated sigma factors, ribosomes, and intracellular metabolites as well as by other interacting global or local TFs (5–12). In addition, previous studies have revealed the extent of TF and growth rate-dependent effects on gene expression and metabolic pathways during exponential growth as well as in the event of adaptive evolution (5, 8). Therefore, to deepen our understanding of regulation prevalent within a transcriptional regulatory network (TRN), it is fundamental to investigate these indirect or growth rate-mediated genes in conjunction with the known functions of the direct targets of the interrogated global TF for a particular environment.

The global TFs which regulate a wide array of genes are known to work in concert with local or pathway-specific TFs that regulate fewer genes, albeit in a less characterized fashion. In Escherichia coli, it is estimated that around 51% of the gene expression changes are modulated by the global TFs (CRP, FNR, ArcA, IHF, HNS, Fis, and Lrp), while the remaining 49% are driven by their interaction with other pathway-specific local TFs (3, 13). As gene expression can be controlled by more than two regulators, it becomes crucial to determine the mode of interaction between the global TFs and other interacting TFs within the TRN. These interactions could quite often represent epistatic interactions rather than direct protein-protein interactions. Deciphering whether such epistatic interactions in the TRN are due to changes in gene expression of their interacting TFs or metabolite-driven changes in their TF activities remains fundamental.

In a canonical sense, the transcript levels majorly dictate the abundance of enzymes followed by changes in metabolic flux and metabolite levels (14–17). *In vivo* studies concerning the allosteric effects of metabolites on TFs are now gaining importance, as they further enhance our understanding of metabolism (15, 18). Identifying such metabolite-TF interactions during batch exponential growth based on steady-state physiological concentrations of the intracellular metabolites and activities of the TFs has been difficult. However, even a simple correlation analysis of the metabolite concentrations with the inferred TF activities based on gene expression data will provide insights into the plausible metabolite-TF interactions, which can then be verified experimentally. Apart from the metabolite-TF interactions, other modes of growth rate-dependent regulation can control the expression of the indirect gene targets. Further, questions concerning the expression of specific sets of indirect genes and their connection with direct targets remain unanswered. Therefore, deconvoluting the complexity of gene expression in terms of coexpression as well as identifying the clusters of directly coregulated genes becomes imperative (19). Given the availability of enormous gene expression data sets for E. coli K-12 under various nutritional or stress conditions (20), it would be a valuable resource to derive the conserved coexpressed genes. However, the analysis of coexpressed genes in the context of maintaining the demand-and-supply equilibrium of intracellular metabolites remains largely unexplored.

**FIG 1:**
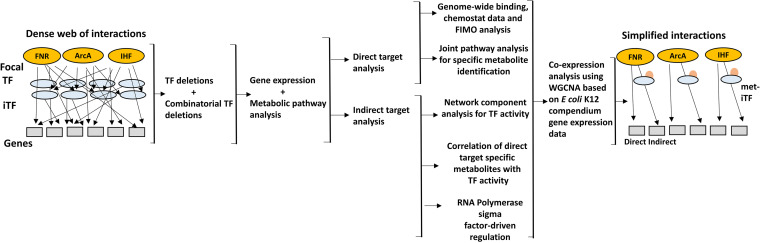
Overview of the integrated workflow to dissect the complex regulation of gene expression within the TRN. Analyses were carried out for the global regulators FNR, ArcA, and IHF by employing single or combinatorial gene deletions under anaerobic glucose-fermentative conditions. Yellow ovals represent global TFs, blue ovals represent other TFs (global or local), orange ovals represent metabolites, gray boxes represent direct and indirect metabolic gene targets, and arrows represent regulation (positive or negative).

Here, we sought to investigate the complex layers of regulation driven by the global TFs using single and combinatorial knockouts of FNR, ArcA, and IHF regulators under glucose-fermentative conditions. FNR and ArcA are best studied for their roles during anaerobiosis and energy metabolism (21–26). Similarly, IHF was been well characterized for its functions as a nucleoid protein as well as its role in central carbon metabolism (26–28). In this study, we examined these global regulators with a focus on direct and indirect targets, epistatic interactions with other TFs, and their preferences for specific metabolic genes in order to formulate an appropriate response. To this end, we utilized an integrated framework that disentangled the pleiotropic regulatory responses for a particular environment (Fig. 1). First, we determined the gene expression profiles and metabolite levels in the strains lacking these global TFs, followed by extensive computational analyses. We delineated the direct and indirect target genes and inferred the regulator activities using network component analysis (NCA) (29) and the corresponding metabolite-TF interactions, which together gave us insights into the regulator-driven epistatic interactions within the TRN. Moreover, we elucidated the coordination between the direct and indirectly coregulated genes by employing weighted gene coexpression network analysis (WGCNA) (30–32) on E. coli K-12 compendium gene expression data. Furthermore, we elucidated the cross talk between these global TFs using their combinatorial deletions to decipher whether they exhibit additive or nonadditive effects on the genes that they regulate. Overall, our study can serve as a template for understanding systems-level regulatory events and may even be applicable for deciphering the mechanistic strategies developed during pathogenesis, gut microbiome interactions, and cancer metabolism.

## RESULTS

### Cross talk and nature of interaction within FNR, ArcA, and IHF regulatory networks.

**FIG 2:**
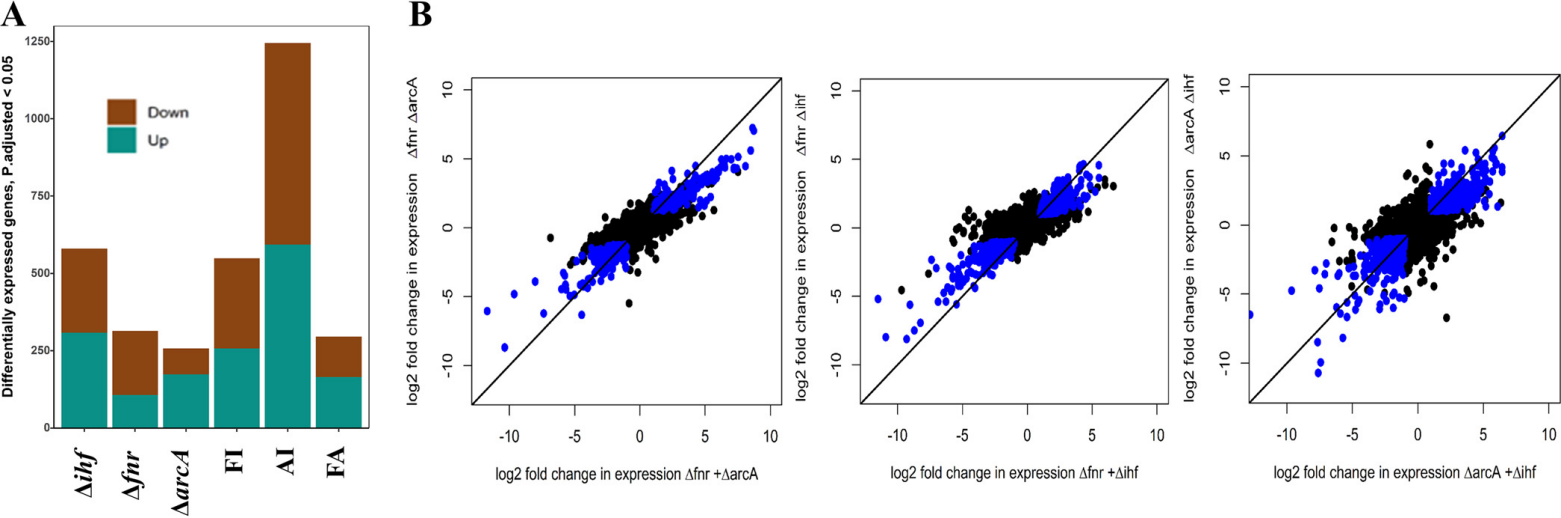
Transcriptome comparison of the regulator mutants compared to the WT. (A) Bar plot showing the numbers of upregulated and downregulated DEGs in the single and double mutants compared to the WT. (B) Scatterplot depicting the mode of interaction in FNR, ArcA, and IHF regulators using log_2_ fold changes in the double mutant compared to the sum of log_2_ fold changes in the single mutants. The blue dots represent DEGs in double mutants (≥2-fold) and genes having a change of ≥2-fold by summing the log_2_ fold changes in each of its single mutants. The black dots represent either non-DEGs or genes that do not satisfy the above criteria.

We constructed three double mutants, FA (Δ*fnr* Δ*arcA*), FI (Δ*fnr* Δ*ihf*), and AI (Δ*arcA Δihf*), by deleting *arcA*, *fnr*, and *ihf* (both *ihfA* and *ihfB*) genes in combination, using homologous recombination (33). We monitored the gene expression profiles for the double mutants compared to the wild type (WT) in the mid-exponential phase under glucose-fermentative conditions. The gene expression data for the FA mutant were found to be consistent with a previous study (34) (see Data Set S1 in the supplemental material). However, gene expression in the mutants FI and AI had not previously been characterized. The gene expression data for the single mutants were obtained from our previous study (26). We investigated the mode of interaction between each of the regulators in the double mutant—specifically, whether the expression patterns could be explained by either of the deleted TFs or by combined pleiotropic effects of both the regulators, defined here as cross talk. Preliminary analysis was performed for both the double and single mutants using the total number of differentially expressed genes (DEGs) (*P* < 0.05, Benjamini-Hochberg [BH] adjusted, log_2_ fold change ≥ |1|). We observed ~250, ~510, and ~1,250 DEGs in FA, FI, and AI, respectively, compared to the WT. In FA and FI, the number of DEGs was lower than in their single mutants combined (Fig. 2A). This was in contrast to AI, wherein the number of DEGs was greater than the sum of the DEGs in its single mutants. This indicated a greater extent of cross talk between ArcA and IHF regulators than in combinations involving FNR.

To understand the exact nature of this cross talk as well as to gain quantitative insights, we first assessed whether the DEGs in the single mutants reproduced their gene expression patterns in the double mutants. We found that the genes whose expression was significantly downregulated or upregulated (DEGs) in the single mutants were also downregulated or upregulated in the double mutant (log_2_ fold change < 0 or > 0) (Fig. S1 and S2). Next, we plotted the genes which were differentially expressed in the double mutant but showed a >2-fold change in gene expression in both single knockouts added together (Fig. 2B). The results indicated that either of the single mutants could explain the observed changes in the double mutants, which provided direct evidence of widespread additive effects between the regulators. We quantified the effects of each of the regulator deletions in the double mutants and performed a two-proportion Z-test with Yates’ continuity correction for assessing significance (*P* < 0.01) (Data Set S1). In the DEGs of the double mutants, upregulated or downregulated gene expression changes could be attributed to specific regulator effects rather than a collective effect. In addition, the genes which could not be assigned (~20 to 38%) to either regulator deletion effect were significant in the case of AI followed by FA and FI mutants, depicting the extent of cross talk. Among DEGs of the double mutants, we observed only ~11 to 20% overlapping genes of both the single mutants with a >2-fold change in gene expression. Overall, these data indicated that in the double mutants, each of the regulators showed independent but additive cross talk effects due to their deletion.

### Regulatory effects on key metabolic pathways.

**FIG 3:**
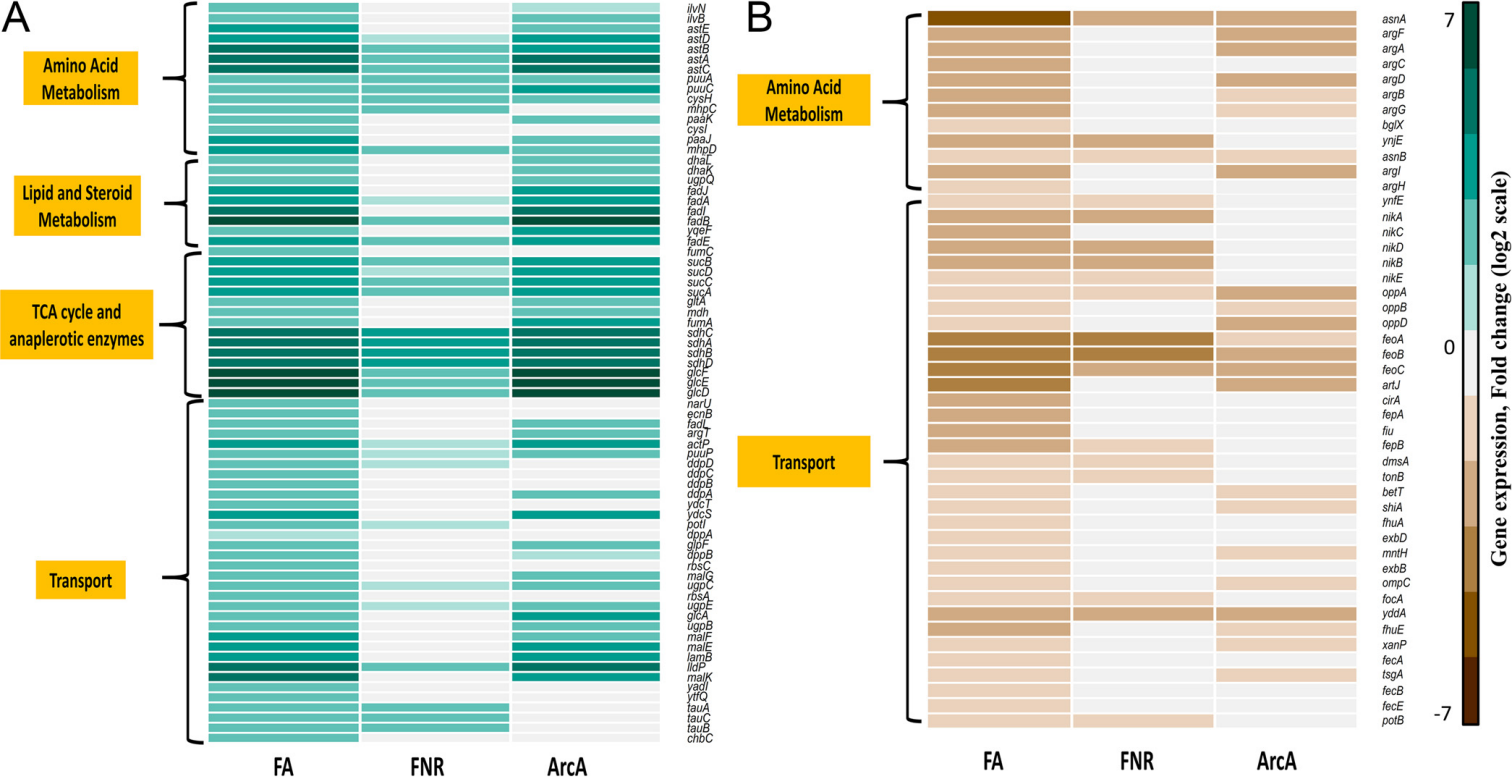
Heat map depicting the metabolic pathway analysis of regulator mutants compared to the WT. DEGs of FA that were (A) upregulated and (B) downregulated compared to the WT are shown. Only the significantly enriched KEGG pathways are shown. For comparison across the strains, only the DEGs found in FA, Δ*fnr*, and Δ*arcA* mutants compared to the WT were considered. See Fig. S3 for metabolic pathway analysis of DEGs in FI and AI.

In our previous work, we demonstrated the system-wide effects of the transcriptional regulators FNR, ArcA, and IHF on key metabolic pathways (26). To decipher whether those metabolic pathways were perturbed in the double mutants as well, we carried out a KEGG pathway enrichment analysis (Proteomaps-based gene classification [35]) on DEGs obtained in double mutants with statistical significance analysis. We found that 69% and 74% of downregulated and upregulated DEGs, respectively, were enriched for metabolic pathways in FA compared to the WT (Data Set S1). In these KEGG pathway-enriched (KPE) upregulated DEGs, we observed significant enrichment of amino acid metabolism related to arginine degradation, which generates internal ammonia, costly tricarboxylic acid (TCA) cycle genes that are unnecessary during anaerobic fermentation, and unneeded lipid and steroid degradation genes (Fig. 3A). Additionally, in KPE-downregulated DEGs, we observed significant enrichment of amino acid metabolism and transport-related genes (Fig. 3B). The amino acid metabolism genes were related to arginine and asparagine biosynthesis, whereas the transporters were related to alternate carbon sources. Among the DEGs in FI, with respect to the WT, 57% and 58% of downregulated and upregulated DEGs, respectively, showed enrichment for metabolic pathways (Data Set S1). Only transport-encoding genes were upregulated in KEGG pathway-enriched DEGs (Fig. S3A). Alternatively, the KEGG pathway-downregulated DEGs (Fig. S3B) showed enrichment for metabolism related to branched-chain-amino-acid biosynthesis, glycolysis, and chaperone-mediated protein folding pathways. On the other hand, 53% and 66% of downregulated and upregulated DEGs, respectively, showed enrichment of metabolic pathways in AI compared to the WT (Data Set S1). Amino acid metabolism genes related to putrescine and arginine degradation, aerobic oxidative phosphorylation genes and TCA cycle genes, transporters, and several transcription factors were upregulated in KEGG pathway-enriched DEGs in AI compared to the WT (Fig. S3C to E). We obtained enrichment for amino acid metabolism related to branched-chain amino acid biosynthesis and bacterial motility proteins in downregulated KEGG pathway-enriched DEGs in AI compared to the WT (Fig. S3F). These data, which indicate significant perturbations of metabolic pathways in the double mutants, were consistent with the observations in single mutants. For instance, the metabolic pathway analysis in FA showed dominant effects of ArcA regulation, whereas both FI and AI were strongly inclined toward IHF-based regulation. In addition, the pattern of enrichment was also consistent with previous observations in the case of single mutants, wherein the necessary metabolic pathways of amino acid and nucleotide metabolism were downregulated and unnecessary metabolic pathways such as the aerobic TCA cycle, oxidative phosphorylation, and alternate carbon transporters were upregulated.

### Loss of TFs results in changes in growth rates and metabolite levels.

We monitored the physiological effects—namely, glucose uptake, growth rate, and secretion profiles—of mixed acid fermentation products as a result of the deletion of TFs. We observed detrimental effects on growth rate in all the double mutants relative to the WT (Table S1). The mutants FA, FI, and AI showed reductions of 40%, 30%, and 37% in growth rate, respectively. This reduction in the growth rates was consistent with the severe perturbation seen in the metabolic pathways. The glucose uptake reduction in FA and FI mutants strongly correlated with their respective growth rate changes. Interestingly, the reduction in glucose uptake in AI was only 13% compared to the WT, as opposed to the Δ*arcA* and Δ*ihf* mutants, despite severe reduction in its growth rate. A severe reduction in biomass yield was also observed in the case of AI (23%), indicating reduced carbon partitioning toward biomass (Table S1). The secretion profiles of exo-metabolites, namely, ethanol, acetate, formate, lactate, and succinate, revealed distinct changes in their yields. Toward a detailed understanding of metabolism, we monitored the intracellular metabolite concentrations for the glucose fermentative metabolism in all the mutants using ^13^C-labeled targeted metabolomics (8, 26). Among the measured metabolites, the intracellular concentrations of glycolytic and TCA cycle intermediates and proteinogenic amino acids were significantly different compared to those in the WT (Student’s *t* test, FDR < 0.05). In FI and AI, we observed increased accumulation of the metabolic precursors and amino acids consistent with the accumulations seen in the single mutants, as reported previously (26) (Fig. S4). These accumulations reflected the inability of the mutants to utilize the metabolites for biomass synthesis, in agreement with their reduced growth rates. However, we observed lower pool sizes of these metabolites in the FA mutant than in the WT. This might indicate an adverse carbon limitation (36) as well as the reduced availability of active ribosome pools (37–42) prevalent in slow-growth conditions, which needs to be investigated in the future.

### Dissecting direct and indirect targets of the TF from their pleiotropic effects on metabolism.

We sought to disentangle the growth rate-mediated indirect effects from the direct effects arising from the absence of FNR, ArcA, and IHF regulators and their pairwise combinations, referred to here as focal TFs. Here, we employed a multipronged approach using the growth rate-dependent genes obtained from chemostat experiments, genome-wide studies to enrich for the binding region of the regulators and comparison with available gene expression data to account for all the genes modulated purely by the focal TFs. First, we performed glucose-limited chemostat cultivations of the WT strain under aerobic respiratory and anaerobic fermentative conditions at a fixed dilution rate of 0.21 h^−1^ to identify the genes whose expression changes were growth rate independent. A comparison of DEGs in the WT in anaerobic fermentation relative to the WT under aerobic conditions highlighted the genes that are specific to regulation or genes that are not altered due to slow-growth effects during anaerobic fermentation. It was shown previously that the gene expression changes in response to growth rate effects are consistent, despite glucose-limited or excess-glucose conditions (5, 8, 9). Thus, the DEGs identified under the chemostat conditions were then utilized to characterize the indirect targets as growth rate-mediated effects and the direct targets as regulation-specific effects by the focal TF(s) under batch exponential growth conditions. The DEGs enriched for direct targets of the focal TFs in the double mutants were further validated by determining the binding motifs using FIMO analysis (43) together with the available *in vivo* chromatin immunoprecipitation (ChIP) binding or other *in vitro* binding data (22–25, 27, 44, 45). Further, a few regulation-specific genes (15 to 20% in double mutants and 20 to 32% in single mutants) (Data Set S1) found in our analysis that were not characterized as direct targets could potentially represent uncharacterized novel targets (46) of the focal TF(s).

**FIG 4:**
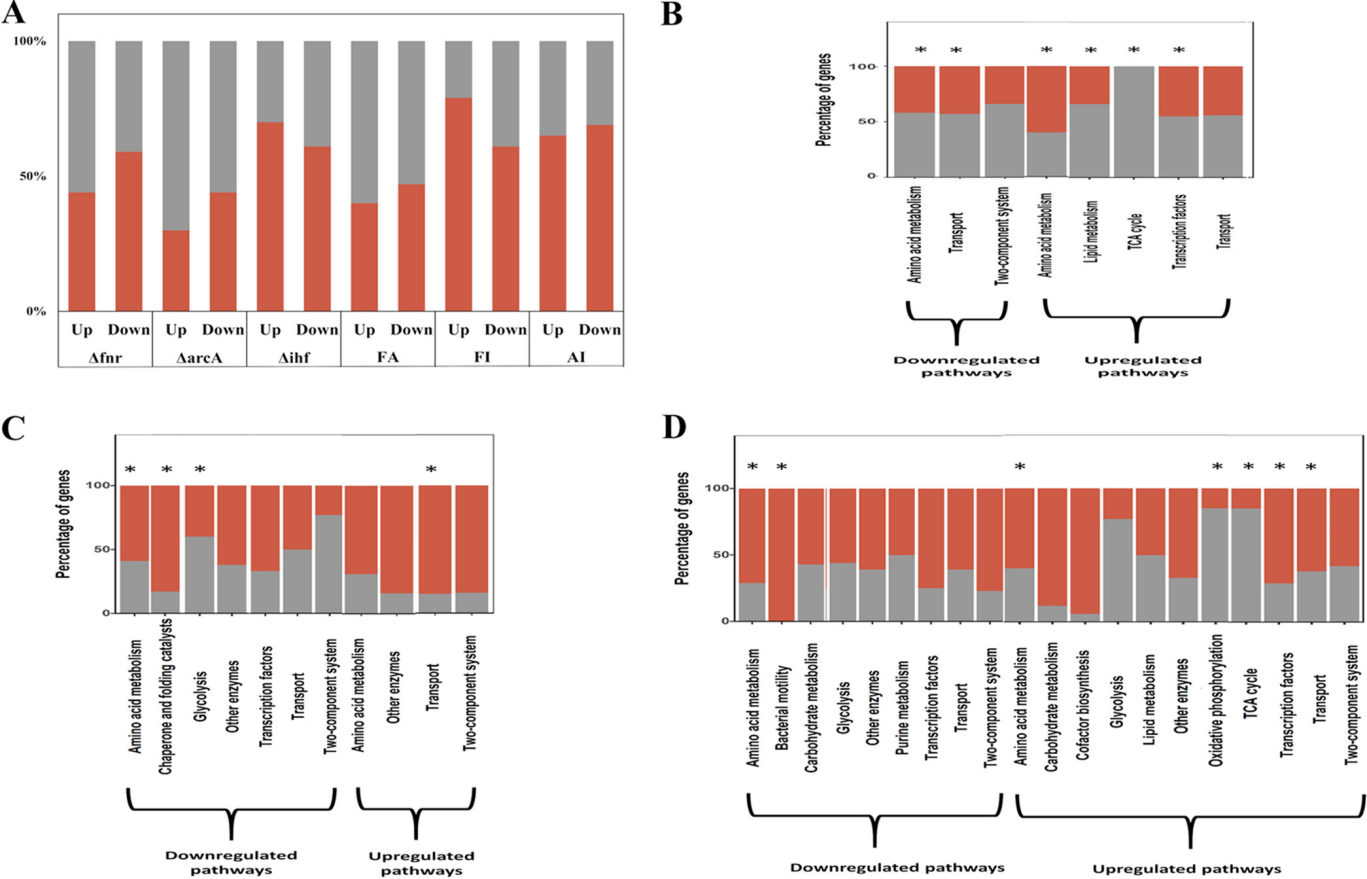
Direct and indirect effects on gene expression. Stacked plots depicting the percentage of direct targets and growth rate-mediated indirect targets (A) in KPE DEGs, as a result of deletion of the focal TFs, (B) FA, (C) FI, and (D) AI compared to the WT. Gray sections of the bars represent regulation-specific effects, and orange sections represent growth rate-mediated effects. Asterisks indicate the significantly enriched metabolic pathways. Only the metabolic pathways which had at least 9 genes were retained for the analysis. See Data Set S1 for the complete list. See Fig. S5 for the single-TF-deletion mutants.

We delineated the direct regulation-specific or indirect growth-rate mediated effects on metabolism by taking into consideration the KPE DEGs. Overall, we found that 55 to 60% of upregulated and downregulated KPE DEGs were regulation specific in FA, compared to 30 to 33% in FI and AI (Fig. 4A). The slow-growth-mediated changes in gene expression were dominant in the FI and AI mutants. In the case of single mutants, the proportions of indirect genes were most prominent in the Δ*ihf* mutant, followed by the Δ*fnr* and Δ*arcA* mutants. The significantly enriched pathways within the upregulated KPE DEGs in the double mutants (except FI) as well as the single mutants (except the Δ*ihf* mutant) showed a greater extent of regulation-specific effects. However, the data did not ascertain whether the reduction in growth rate was proportional to the extent of growth rate-mediated or indirect targets. On the other hand, the significantly enriched pathways within the downregulated KPE DEGs of the strains (except FA) aligned well with the reduced growth rate (Fig. 4B to D; Fig. S5A to C).

### Exploring the effects of regulator-driven epistatic interactions on indirect target expression.

To understand the epistatic interactions of the focal TF with other global or local TFs, we considered that the enrichment of direct targets majorly responded to the focal TF(s). In addition, we assumed that the probability of regulating the indirect targets by the focal TF is low, as they might represent silent targets (23, 46) during exponential growth under glucose-fermentative condition. Silent targets represent genes which, despite having upstream binding sites for the focal TF, are not regulated under this specific condition. This could be due to either a masking effect by other TFs or a cooperative effect of multiple TFs or condition-specific regulation. Hence, we analyzed the indirect gene targets for any TF enrichment other than the focal TF. These regulated TFs are epistatically interacting TFs (iTFs) whose gene expression or activity is modulated by the focal TF(s). The enrichment of genes for the presence of binding of iTFs was done using EcoCyc or RegulonDB (44, 45) and further assessed for statistical significance (Fisher’s exact test, FDR < 0.05). The KPE DEGs of each of the mutants were used as an initial set to characterize all the possible iTF enrichments (Table 1). Classification of the KPE DEGs based on our approach indicated (i) a drastic reduction in spurious interactions as observed from iTF enrichment on indirect targets (Table 1) and (ii) an increased number of direct targets for each of the focal TFs (Data Set S1). The activity of the iTFs was then determined based on the manual interpretation of the changes in their target gene expression (Data Set S1). We found that in most cases, changes in inferred activity of iTFs were either not correspondent or antagonistically correspondent with the changes in their gene expression. Only MhpR in AI, Nac in FI, and ArcA in the Δ*ihf* mutant showed an increase in their gene expression concomitant with their increased activities (Table 1). Overall, assessment of the changes in gene expressions of the iTFs itself revealed that the inferred activity of the iTFs was perturbed for reasons not involving its gene expression. The other possible mechanisms involve changes at the posttranslational level or the protein level. As the transcript abundances majorly dictate the protein concentrations (47, 48), we further investigated the focal TF-iTF epistatic interaction at the posttranslational level.

**TABLE 1:** iTFs for each of the focal TF mutants on the KEGG pathway-enriched DEGs^*a*^

Mutation or strain	Regulation	iTF showing^*b*^:	
		Epistatic interactions for each focal TF mutant^*c*^	True epistatic interactions using our approach^*d*^
Δ*fnr*	Up	ArcA, IHF, Fur, PuuR, CysB, NtrC, ArgR	**ArcA**, **PuuR**, NtrC, Crp
	Down	IHF, Fur, Crp, Fis, NrdR, GlpR, NagC, RstA, NarL	
Δ*arcA*	Up	Crp, IHF, Fis, FNR, PuuR, NarL, FadR, PhoB, MalT, GatR, GlcC	**CRP, PuuR**, IHF
	Down	Fur, Cra, ArgR, AppY, YdeO, MetJ, IscR, ModE	
Δ*ihf*	Up	CysB, TrpR, HypT, UlaR	**CysB, TrpR, UlaR**
	Down	ArcA, HNS, Fur, Lrp, Cra, OmpR, FliZ, GadE, Nac, RcsB, PuuR, CsgD, RcdA, NrdR, GadX	HNS, **Lrp, ArcA, OmpR, PuuR**
FA	Up	CRP, IHF, NtrC, MalT, FadR, CysB, PhoB, ArgR, PuuR, GlcC, PdhR, PrpR	**CRP, PuuR, NtrC**
	Down	Fur, NarL, Cra, NrdR, HypT, NikR, ArgR, PdhR	**Fur**, PdhR
FI	Up	CysB, Nac	**CysB, Nac**
	Down	CRP, Fur, Lrp, ArcA, Fis, HNS, Cra, NarL, ModE, RstA, GadE, Nac, NrdR, OmpR, FliZ, IscR, MlrA	**Fur, Lrp**
AI	Up	CRP, FNR, NarL, NtrC, CysB, ArgR, NarP, ModE, FadR, PuuR, MetJ, TrpR, UlaR, MhpR, RutR, DcuR, GalR, GalS, GatR	**NtrC, CysB, PuuR, UlaR, MetJ, TrpR, MhpR**, RutR, ***BirA***
	Down	Fur, HNS, Lrp, RcsB, PhoB, OmpR, Nac, GadE, CsgD, CusR, HprR, GadX, FliZ, NrdR, RcsB-BglJ, HypT	HNS, **Fur, Lrp, PhoB**, RcsB, OmpR, Nac, **CusR, HprR**, CsgD, RcsB-BglJ, HypT

a The genes enriched for focal TF itself are not shown here for simplicity.

b The iTFs in bold are those that show correlation with metabolite, do not show dual regulation, or are successfully reproduced from NCA analysis, which were further utilized for determining conserved metabolite-iTF interactions seen across all the mutants (Fig. 5A). The underlined iTFs are those whose gene expression matches their activity changes. The iTF in italics represents a new iTF not found in the complete list mentioned in the third column.

c Complete list of iTFs (iTF enrichment on upregulated and downregulated genes) without any categorization.

d Final iTFs (iTF enrichment for indirect targets) after categorization using our approach.

The metabolic precursors and amino acids, apart from being building blocks for protein biomass, can also act as signaling molecules that can allosterically modulate the activities of the TFs to bring about changes in the gene expression of the organism (14, 15, 17, 49, 50). The concept used to define focal TF-iTF epistatic interaction was that metabolites perturbed due to changes in direct targets by focal TF(s) allosterically regulate the iTFs, which then modulated the indirect or growth rate-mediated gene targets.

To address the putative allosteric metabolite-iTF interactions, we carried out a linear correlation analysis (Pearson correlation coefficient [*r*] > 0.75, *P* < 0.05) between the KPE direct-target-specific metabolite concentrations and the iTF activities. As determination of metabolite-iTF interactions by linear correlation is prone to high numbers of false positives, to increase the confidence of such putative metabolite-iTF interactions, two stringent criteria were applied. First, only metabolites that were perturbed due to the direct targets of focal TFs were considered. To obtain the list of metabolites, a joint pathway analysis was performed using the significantly perturbed metabolites with the combined upregulated and downregulated KPE direct targets for each strain separately and the metabolite-gene pair which satisfied the minimum threshold (see Materials and Methods). The metabolites that responded to changes in direct targets were mainly glutamate, aspartate, phosphoenolpyruvate (PEP), α-ketoglutaric acid (αKG), γ-aminobutyric acid (GABA), citrate, glycine, and to a lesser extent glutamine, arginine, and lysine (Data Set S1). The other metabolites which were significantly (FDR < 0.05) perturbed in each of the strains could be ascribable to the concomitant changes in its precursor metabolites. For instance, changes in methionine levels were concomitant with the changes in its precursor aspartate (Fig. S4). Second, the metabolite-iTF pair was reanalyzed using the pathway information available in EcoCyc to identify whether the metabolite (Pearson correlation coefficient [*r*] > 0.9, *P* < 0.05) is related to the KPE indirect gene targets for that particular iTF. This work is similar to the distance criterion (15) approach, wherein the iTF regulating the gene (that encodes the enzyme) and metabolite are part of the same metabolic subsystem. Additionally, we compared the metabolite-iTF interactions with published studies reporting similar putative metabolite-TF interactions (15).

To quantify the iTF activities, we used the network component analysis (NCA) algorithm (29), which derives the regulatory activity from the experimentally measured gene expression profiles. For each of the strains, NCA was performed separately on the direct or indirect KPE DEGs. With NCA, we were able to reproduce 80% of the iTF activities for each of the strains, including the known interactions as well as novel putative metabolite-iTF interactions (Fig. S6A to F). For instance, we were able to reproduce the known interactions of PuuR with putrescine (inferred from GABA [26]) as well as CRP with cAMP (inferred from PEP [8, 51]) (Fig. 5A and B). The metabolite cAMP was not detectable in our liquid chromatography-mass spectrometry (LC-MS) run, probably due to the limit of detection and anaerobic fermentation conditions with high glucose uptake rate (it was detectable under aerobic conditions). As cAMP and PEP levels are interdependent (52, 53), we assumed the association of CRP with PEP, similar to its association with cAMP. For FA, we inferred increased activity of CRP, NtrC, and Fur and decreased activity of PuuR along with their allosteric effectors, such as PEP, αKG, citrate, GABA, glycine, and arginine (Data Set S1). This inference was consistent with increased activity of CRP with its interacting metabolites PEP, αKG, and citrate in the Δ*arcA* mutant and NtrC in the Δ*fnr* mutant as well as decreased activity of PuuR with its allosteric metabolites GABA, PEP, and αKG in both single mutants. In the FI mutant, we inferred increased activity of Fur, CysB, and Nac and decreased activity of Lrp. The inferred activity changes of CysB and Lrp along with their interactions with metabolites GABA, arginine, glutamate, αKG, and PEP were consistent with their inferred activity profile observed in the Δ*ihf* mutant. For AI, we inferred a large number of regulators with changes in their activity profiles, concomitant with the widespread changes in the gene expression profiles, which reinforces the greater cross talk effects between ArcA and IHF regulators. The decreased activity of Lrp, PuuR, TrpR, and UlaR and the increased activity of CysB along with their interactions with glutamine, glutamate, aspartate, PEP, arginine, and GABA in the AI mutant was consistent with the inferred activity profiles of Lrp, CysB, TrpR, and UlaR in the Δ*ihf* mutant and PuuR in the Δ*arcA* mutant.

**FIG 5:**
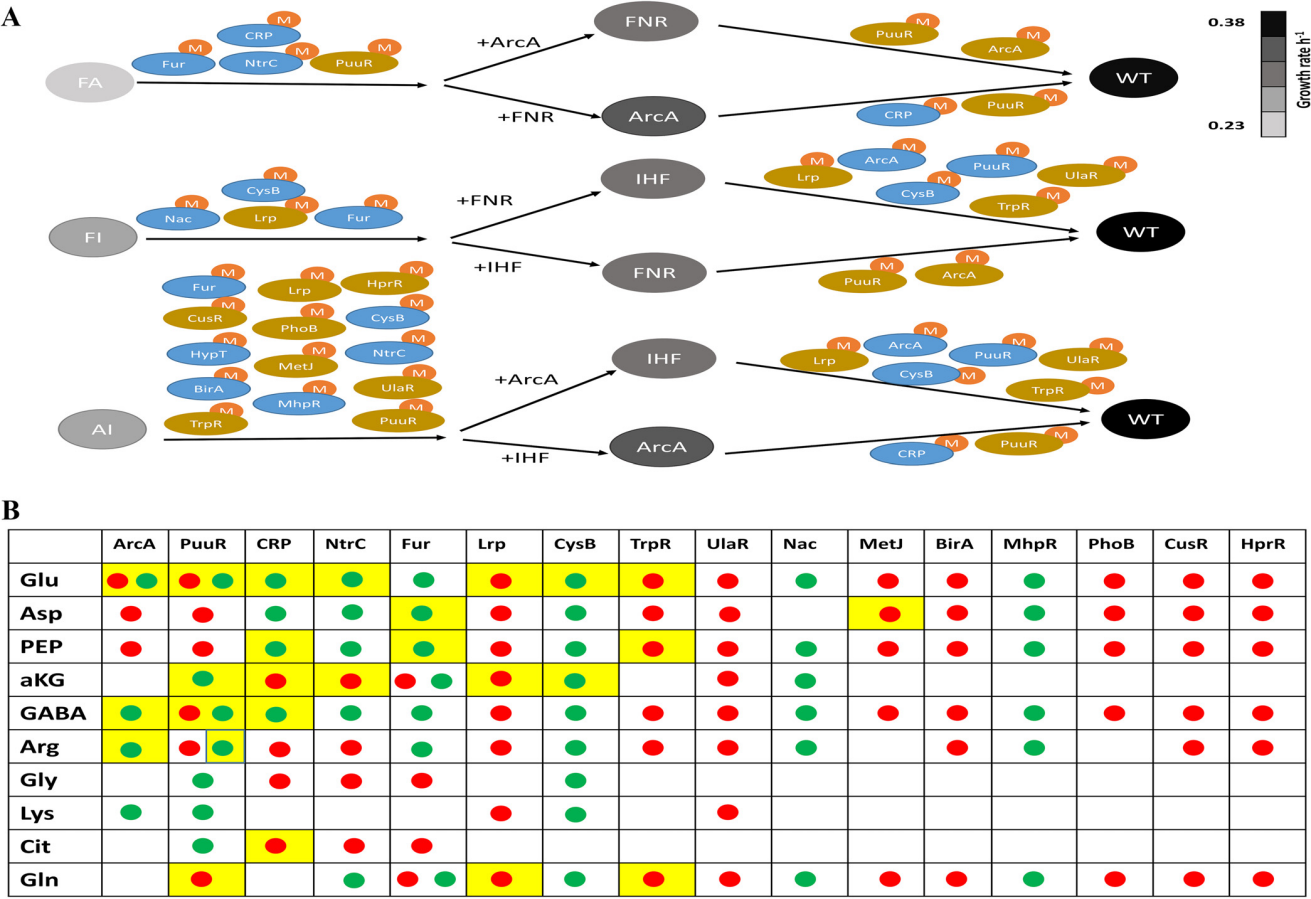
Characterization of conserved and novel metabolite-TF interactions in each double mutant and its corresponding single mutants. (A) M, metabolites (characterized in panel B) that allosterically modulate the inferred iTF activity. iTFs with increased and decreased activity in the absence of the focal TFs are depicted with blue and brown, respectively. The color of the double and single mutants is based on their specific growth rates. (B) Comprehensive identification of putative metabolite-iTF interactions using the Pearson correlation (*P* < 0.05) of metabolite concentrations with inferred iTF activity observed in each of the strains. The green and red dots represent positive and negative correlations, respectively. A subset of putative but high-confidence metabolite-iTF interactions with Pearson correlation (*r* > 0.9, *P* < 0.01) that belong to the same metabolic subsystem where this iTF regulates as well as putative interactions reported in the literature are highlighted in yellow. Glu, Glutamate; Asp, Aspartate; PEP, phosphoenolpyruvate; aKG, α-ketoglutarate; GABA, γ-aminobutyric acid; Arg, arginine; Gly, glycine; Lys, lysine; Cit, citrate; Gln, glutamine.

Next, we examined across the strains the metabolite-iTF interactions as a function of growth rate (Fig. 5A). This analysis revealed not only the shared patterns of interactions seen in the double and single mutants but also the cross talk effects. For instance, metabolite-ArcA interactions were more prominent at higher growth rates, as opposed to metabolite-Fur interactions, which were seen with lower growth rates. AI showed the highest number of metabolite-TF interactions compared to its single mutants, which reiterated the cross talk effects, in contrast to the interactions seen in FA and FI mutants. Based on all the metabolite-iTF interactions across the strains, we wanted to determine the conserved metabolite-iTF pairs. In total, we observed 119 connections between 10 metabolites and 16 iTFs (Fig. 5B). We found that 12 metabolite-iTF connections which showed positive as well as negative associations (glutamate-ArcA, glutamate/GABA/arginine-PuuR and αKG/glutamine-Fur) accounted for only 10% of the metabolite-iTF connections, whereas the remaining 90% were unique. We observed that glutamate, aspartate, PEP, and GABA had the greatest number of allosteric connections with iTFs. These were followed by arginine, α-KG, and glutamine with medium connectivity and glycine, lysine, and citrate with low connectivity with their respective iTFs. A total of 29 connections with 9 iTFs and 8 metabolites were observed (Fig. 5B). Among these, ~45% of the interactions/associations were reported in a previous study (15). Overall, we were able to determine with high confidence novel metabolite-TF interactions that provided insights into the regulatory interactions within the TRN (Data Set S1).

### Coexpression analysis of the direct and indirect KPE DEGs.

Though a significant proportion (30%) of the indirect genes reflected the metabolite-driven allosteric regulation, a higher proportion (70%) of the genes did not show any significant (Fisher’s exact test, *P* < 0.05, BH adjusted) enrichment for any iTF binding (Data Set S1). This remaining set of genes was annotated as being predominantly regulated by RNA polymerase-associated sigma factors either by using EcoCyc or based on promoter prediction using iPromoter-2L (54, 55) (Data Set S1). RNA polymerase-associated sigma factors were used because they are known to constitute an indirect readout of growth rate-dependent global machinery (6, 7, 11). Further, to gain a systematic understanding of the coordinated changes in direct targets and indirect targets, including those driven by the underlying metabolite-iTF interaction, we utilized WGCNA (31), an unsupervised technique to cluster genes using correlation of their expression profiles. Such analyses can also reveal the coregulated genes directly modulated by the focal TF(s), which has been a major challenge in understanding the regulatory principles (19, 56). We employed a large-scale compendium of gene expression data (COLOMBOS) (20) comprising microarray and RNA sequencing data as an input for generating the network of coexpressed genes in E. coli (Fig. S7A and B). Among 4,077 contrasts and 4,189 genes used for the analysis, we observed occurrences of 7 coexpression merged modules (Data Set S1). Among these modules, blue (1,274) and turquoise (1,572) covered the majority (~68%) of the genes. We performed gene ontology (GO) analysis to identify the functions of the KPE genes falling in a particular module for both the direct and indirect upregulated and downregulated genes in each of the mutants.

**FIG 6:**
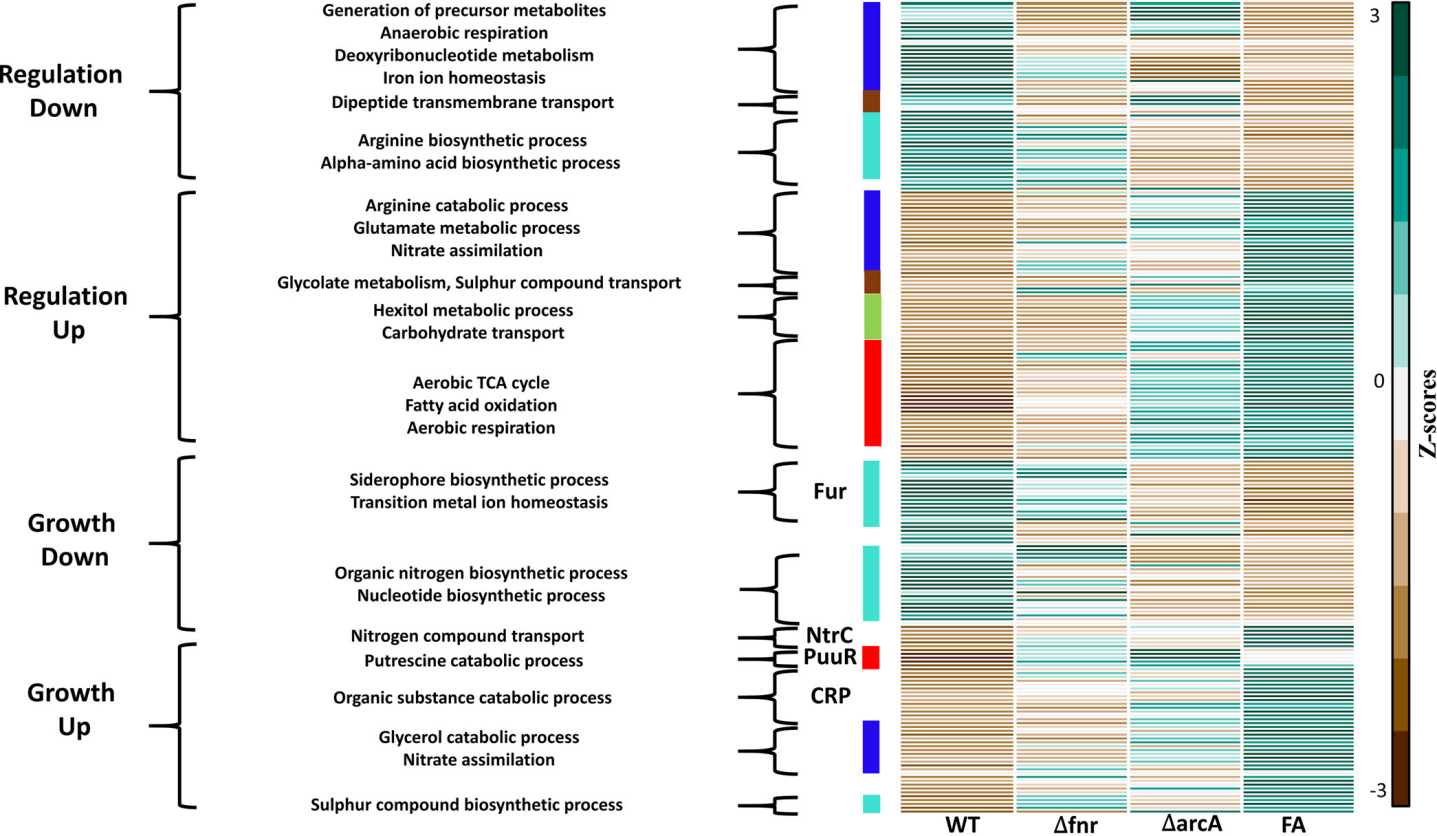
Heat map depicting the regulation-specific direct and growth-rate mediated indirect targets for FA compared to the WT. KPE DEGs that were upregulated and downregulated in the FA mutant compared to the WT were used to compare across the strains. The values (transcripts per million) calculated for each double mutant and corresponding single mutants were Z-score transformed. The bars next to the heat map represent the different coexpression modules, displayed in different colors. Also, all iTFs enriched from the indirect targets are represented along with the functions of the downstream targets. The GO categories or TFs that have genes belonging to more than 1 module color are shown as blank regions between the colored bars. See Fig. S8A to D for FI and AI.

We identified the coregulated genes and their functions controlled by the regulators FNR, ArcA, and IHF, as indicated by the module colors. Further, the GO categories enriched for each of the modules in the direct targets were found to be similar to the enrichments seen in the indirect targets (Fig. 6 and Fig. S8A to D). Across the mutants, we found enrichment for generation of precursor metabolites, deoxyribonucleotide metabolism, or protein transport in the blue module in the downregulated direct targets that showed coexpression with negative regulation of macromolecule biosynthesis in the blue module of downregulated indirect targets. Such coexpression patterns between the direct and indirect genes underscore the constraints on precursor or metabolite usage. Similarly, we found enrichment for the α-amino acid metabolic process, the arginine-biosynthetic process, or the glutamate metabolic process in the turquoise module in the downregulated direct targets that showed coexpression with the cellular compound nitrogen biosynthetic process in the turquoise module of downregulated indirect targets. Moreover, in the case of the red module of upregulated direct and indirect targets, we found coexpression of genes enriched for the TCA cycle, fatty acid metabolism, or aerobic respiration with the putrescine catabolic process in most of the mutants. This reflected the regeneration of αKG through putrescine degradation, as the upregulation of the aerobic TCA cycle genes can result in its futile cycling. Overall, if the direct effects of the focal TFs resulted in changes in the biosynthetic process, the metabolite-driven iTFs concomitantly regulated alternative biosynthetic genes (Data Set S1), thereby regulating the economy of the metabolite.

## DISCUSSION

Bacteria like E. coli have developed regulatory strategies coordinated by TFs and growth rate-dependent global machinery to sense and respond to environmental cues. To gain a clear understanding of the regulatory logic, delineating the TF-driven pleiotropic effects that exist within the TRN becomes imperative. Using our system-level framework, we deciphered (i) direct and indirect pleiotropic effects of the focal TFs, (ii) epistatic interactions between the focal TFs and iTFs, (iii) high-confidence putative metabolite-iTF interactions, and (iv) coordination between the direct and indirect target expression that is related to the conditional regulation by specific iTFs of downstream genes. We evaluated these objectives using multi-omics data derived from E. coli strains with combinatorial deletions of FNR, ArcA, and IHF regulators under glucose-fermentative conditions. Consistent with our previous work that explained the individual effects of each of the focal TFs at the gene level (26), we found that each double-deletion mutant of the focal TFs resulted in downregulation of necessary metabolic pathways and upregulation of unnecessary ones. Despite the similarity at the pathway level, individual genes were modulated by specific focal TFs in each double mutant rather than collectively. Such results indicated the independent yet additive mode of epistatic interactions between the global regulators, with more cross talk effects seen in the combination involving ArcA and IHF. Overall, by employing a combinatorial deletion approach, this study provides a paradigm for identifying the conserved regulatory pattern across the mutants, the amount of overlap in genes and functions, and target expression specificity arising out of the mode of epistatic interactions of the TFs.

Using our integrated approach, we found that across the strains lacking FNR, ArcA, or IHF and their combinations, a greater percentage (>45%) of DEGs were associated with the direct effects of the focal TF than was estimated earlier (~17%) (26). This was determined from the available genome-wide TF binding data, from the detection of novel genes wherein the upstream DNA binding sequence was predicted with good confidence, and from chemostat-based gene expression analyses. Further, we identified the indirect gene targets under batch fermentative conditions for each of the TF deletion strains. These indirect targets of the focal TFs were determined from the gene sets modulated by growth rate in terms of RNA polymerase sigma factor distribution as well as the interactions with other iTFs based on metabolite regulation. Functionally, both the direct and indirect targets of the focal TFs represented not only the metabolic bottleneck genes but also hedging or alternate carbon metabolism genes that can facilitate growth under new environmental conditions. Last, we inferred that the distribution of the sigma factors across the indirect genes could also explain their gene expression changes in response to nutrient limitations (largely carbon and nitrogen and to a lesser extent phosphate or sulfur) as a result of reduced growth rates (12, 57, 58).

Despite the overriding complexity within the TRN, our findings demonstrated the nature of focal TF-iTF epistatic interactions and their effect on regulation of gene expression. We found that a majority of the gene expression changes modulated by the enriched iTFs could be ascribable to their posttranslational effects, highlighting the metabolite-driven regulation. Among the total putative metabolite-iTF interactions identified with high confidence in this study, we not only validated the metabolite-iTF interactions (~45%) by comparing the results with published experimental data but also predicted many novel potential metabolite-iTF interactions (~55%). Although we report the putative metabolite-TF interactions using correlation analysis, the stringent criteria used to screen metabolites that (i) responded to changes in direct targets of the focal TF, (ii) were retained after pathway classification, (iii) crossed the threshold correlation value of 0.9, and (iv) were frequently observed across different mutants made these overall high-confidence metabolite-TF associations. Apart from metabolite-based focal TF-iTF interactions, we considered that indirect gene targets that could not be assigned to known iTFs were regulated primarily by RNA polymerase sigma factor. However, it is of particular importance to understand the association between the indirect and direct targets which influence growth physiology. Coexpression analysis using WGCNA revealed the complementary nature of indirect gene targets with direct gene targets in accordance with the metabolite adjustments. For instance, to mitigate the competition for available cellular resources such as precursors and proteinogenic amino acids, if the pathways synthesizing the metabolite is perturbed due to the focal TF, the organism will either reduce the usage of the metabolite in other pathways or promote its degradation if the concentration is found to exceed the demand for the metabolite. Moreover, the coexpressed targets of the focal TF elucidate the preference for interaction with specific iTFs as well as their downstream cognate genes. Hence, we were able to dissect the complexity of regulation on gene expression changes relevant to metabolism. Further, these coexpressed metabolic genes can be considered coregulated genes, given the similar expression profiles and upstream consensus motif sequences for any TF. Overall, the insights obtained from the gene coexpression patterns for E. coli K-12 can now be employed to explore other gene-regulatory mechanisms that enable the organism to cope with stress or changes in carbon sources.

Our previous work showed the allocation of necessary and unnecessary proteome sector and metabolite adjustments, driven by the global transcriptional factors FNR, ArcA, and IHF (26). Here, using an integrated flow of analysis (Fig. 1), we elucidated the plausible regulatory interaction and cross talk effects in formulating an appropriate biological response for a particular environment. However, we note that the correlation analysis explores only the possibility of occurrence of putative metabolite-TF interactions and does not imply direct causation. Thus, additional work involving the experimental validation of metabolite-TF interactions (15, 59) as well as characterizing other modes of regulation, such as small RNA (sRNA) (60) or protein-protein interaction (61), would further contribute to our comprehensive understanding. In addition, the demarcation of the indirect and direct targets of global TFs is condition dependent, meaning that these indirect targets have binding sites for the TF but represent silent targets for that particular environment (23, 46). These sites could also represent relaxed-specificity sites that could enable adaptation to newer environments (62). Additional experiments supporting TF binding to promoters of uncharacterized or putative direct targets will further strengthen our predictions. Nevertheless, this study serves as a valuable approach to accurately deconvolve the complex layers of TF-driven regulation and systems-level regulation, in general.

## MATERIALS AND METHODS

### Strain construction.

The E. coli K-12 strain MG1655 was used to construct the double deletions Δ*fnr* Δ*arcA* (FA), Δ*fnr* Δ*ihf* (FI), and Δ*arcA* Δ*ihf* (AI). Δ*ihf* represents a deletion of the genes encoding both subunits (Table S2). The strains were generated using λRed-mediated homologous recombination using plasmid pKD46, with pKD13 as the template plasmid and pCP20 for removal of markers (33). The gene deletions were verified by PCR as well as Sanger sequencing using primers against the gene of interest. After verifications, stocks were prepared in 25% glycerol (final concentration) and stored at −80°C.

### Media and strain cultivation.

The cells were cultivated anaerobically in a 500-mL bioreactor (Applikon) containing 400 mL of M9 minimal medium (6 g/L anhydrous Na_2_HPO_4_, 3 g/L KH_2_PO_4_, 1 g/L NH_4_Cl, 0.5 g/L NaCl plus 2 mM MgSO_4_ and 0.1 mM CaCl_2_) with 2 g/L glucose as the carbon source. The cultivations and bioreactor settings were done as reported previously (26). Briefly, the cells were used to inoculate the 400 mL of M9 minimal medium with 2 g/L glucose in a bioreactor, and the starting optical density (OD) of the culture was set at approximately 0.07. The temperature in the bioreactor was maintained at 37°C, stirrer speed was 150 rpm, and pH was maintained at 7.2. The bioreactor was sparged with nitrogen to maintain the dissolved oxygen levels at zero. The slope of the linear regression line fit the natural logarithm of the OD_600_ (OD at 600 nm)-versus-time plot; ln(OD_600_) versus time (in hours) was used to calculate the exponential growth rate of the organism. All the physiological and multi-omics characterizations were done in the exponential phase of the organism. The dry cell weight (DCW) for each strain was experimentally derived during its exponential growth phase, where an OD_600_ of 1.0 corresponds to 0.44 g DCW h^−1^.

All phenotypic characterizations were performed in three biological replicates (*n* = 3) as described previously (26). Extracellular supernatants collected during the exponential growth phase were used to determine the rates of glucose uptake as well as the rates of secretion of extracellular mixed acid fermentation metabolites, such as acetate, lactate, pyruvate, succinate, formate, and ethanol, and the yields thereafter. All physiological characterizations were tested for significance by using an unpaired two-tailed Student’s *t* test.

Cell cultivations were performed in a chemostat (5, 8), wherein the feed medium composition was the same as that of the batch medium. The WT strain was grown under anaerobic batch growth until 80% of the maximum biomass was produced (biomass formation was monitored by OD measurements) or until 80% of the glucose present in the medium was consumed (glucose levels were monitored by high-performance liquid chromatography [HPLC]), after which addition of the feed medium was started. The dilution rate in the chemostat was maintained at 0.21 h^−1^. Once the cells reached the steady state, the chemostat cultivation was continued for 3 to 5 residence times, after which the cells were harvested for RNA extraction and sequencing. The RNA sequencing data for aerobic batch growth performed under identical chemostat conditions were obtained from reference (8).

### RNA extraction and mRNA enrichment.

The RNA extraction for each strain was done in the mid-exponential phase for two biological replicates as described previously (26). Library preparation for RNA sequencing was done following a paired-end strand-specific protocol using a NEBNext Ultra Directional RNA library kit. RNA sequencing was carried out on an Illumina HiSeq 4000 system in rapid run mode using the 2 × 150-bp format at Genotypic Technologies, Bangalore, India. For chemostat cultivations (aerobic and anaerobic), the harvested RNA was extracted and sent to C-CAMP NCBS Bangalore for RNA sequencing with a NEBNext Ultra II Directional RNA library kit (single strand specific) and run on an Illumina HiSeq 2500 system (rapid run mode) using the 1 × 50-bp format.

### Transcriptome data analysis.

The raw RNA sequencing files from the mid-exponential phase of batch growth and the chemostat cultivations were first trimmed with CUTADAPT (63) to eliminate the adapter sequences and the low-quality reads. The trimmed reads were mapped to the E. coli K-12 MG1655 genome (GenBank accession number NC_00913.3) using Burrows-Wheeler Aligner (BWA) (64), and the .sam file was then converted to a .bam file using SAMtools (65). Counts at each gene level were assigned with FeatureCounts (66) using the reference genome provided in gene transfer format. The 4,466 genes used for the analysis were obtained from the EcoCyc database (v. 21.5) (44). All rRNA, tRNA, and sRNA genes were excluded from the analysis. Differentially expressed genes were analyzed using EdgeR (67) after eliminating the genes having fewer than 10 reads. The genes that had a ≥2-fold change in gene expression (in both directions) and had a BH-adjusted *P* value of <0.05 were considered DEGs and were used for subsequent analysis. The DEGs were then enriched for metabolic pathways using KEGG pathway classification, and the significance of each pathway was checked using a hypergeometric test (*P* < 0.05, BH adjusted) in R (4.0.3). The criterion for a pathway to be considered significant was at least nine upregulated or downregulated genes. DEGs without any accession ID (EcoCyc), such as phantom genes, were not included in the analysis.

In the comparison of transcriptomes between the WT anaerobic fermentation versus the WT aerobic respiration from glucose-limited chemostat cultivations, the DEGs were annotated as regulation-specific (Data Set S1) and represent genes that are not altered due to changes in growth rate or low growth rate. These DEGs were filtered with the threshold of a (Benjamini-Hochberg-adjusted) *P* value of <0.05 and at least a 2-fold change in expression. These genes were then used to identify the regulation-specific and growth rate-mediated genes in the transcriptome changes of single or double mutants compared to the WT in batch mid-exponential phase. These regulation-specific genes were further validated using previously reported direct gene targets of these regulators from *in vivo* ChIP binding data as well as binding motif prediction analysis using FIMO from the MEME suite (43). The global and pathway-specific local regulators together with their cognate gene targets were obtained from RegulonDB (v 10.6.3) (45). The KPE DEGs were independently enriched for global and local TFs, and significance was assessed by Fisher’s exact test (BH adjusted, *P* < 0.05) with an arbitrary set of 5 genes. It should be noted that even though the deleted TFs can be enriched in the case of indirect gene sets, they were not considered for analysis based on the assumption that these genes have other TF binding sites that overlap or simply that they are bound yet represent silent targets (23, 46) for this particular condition. Further, enriched TFs which have dual regulation were considered neither for NCA nor for correlation analysis. The inferred activity of the iTFs was determined based on the manual interpretation of the changes in its target gene expression. For instance, we defined the activity of an iTF repressor as decreased when there was activation of its gene targets.

Gene ontology analysis was performed using the AmiGO 2 database (68) or topGO package (69). The top categories with either an FDR of <0.05 or a *P* value of <0.05 that accounts for a larger set of genes were considered for further analysis.

### Metabolomics. (i) Extraction of metabolites.

The metabolite extraction for each of the strains was performed in the mid-exponential phase. Metabolite extraction was performed for 3 biological and 2 technical replicates (*n* = 3). A fast-cooling method was employed to quench the harvested cells as described previously (8, 26). ^13^C-labeled extracts were used as internal standards generated by growing WT E. coli K-12 MG1655 cells aerobically and were then used to quantify the key metabolite pool sizes using an isotope-based dilution method as done previously. During the earliest stage of extraction, each of the samples was spiked with a fixed volume of internal standard. The volume of the pooled internal standard was added to each sample such that the external and internal standard peak heights differed less than 5-fold. Following the sample preparation steps (8, 26), the samples were completely dried in a vacuum concentrator and stored at −80°C. Before analysis in an LC-MS/MS instrument, samples were reconstituted in 100 μL of chilled (−20°C) acetonitrile-buffered water (60:40 [vol/vol]) and centrifuged at 4°C for 10 min, and the supernatant was transferred to prechilled glass vials. Buffered water contained 10 mM ammonium acetate (pH 9.23). pH was adjusted using ammonium hydroxide prepared in HPLC-grade water.

### (ii) LC-MS/MS settings.

The samples for metabolomic analysis were run on a high-resolution Orbitrap Q Exactive Plus mass spectrophotometer (Thermo) equipped with a SeQuant ZIC-pHILIC column (Merck; dimensions, 150 mm by 2.1 mm by 5 μm) and in sequence with a ZIC-pHILIC guard column (Merck; dimensions, 20 mm by 2.1 mm by 5 μm) with the LC-MS/MS settings and calibrations as described previously (8, 26). An alkaline mobile phase was used with an electron spray ionization (ESI) ion source operated in positive (M + H)^+^ and negative (M − H)^−^ modes separately. A full scan range of 66.7 to 1000 *m/z* was applied for positive as well as negative modes, and the spectrum data type was set to profile mode. The mobile phase used for chromatographic separation consisted of a nonpolar phase A (acetonitrile-water mixed at a ratio of 9:1, 10 mM ammonium acetate [pH 9.23] using ammonium hydroxide) and polar phase B (acetonitrile-water mixed at a ratio of 1:9, 10 mM ammonium acetate [pH 9.23] using ammonium hydroxide). A linear gradient with a flow rate of 200 μL/min was set as follows: 0 to 1 min, 0% B; 1 to 32 min, 77.5% B; 32 to 36 min, 77.5% B to 100% B; 36 to 40 min, hold at 100% B; 40 to 50 min, 100% B to 0% B; 50 to 65 min, re-equilibration with 0% B. An injection volume of 5 μL was used for all the samples and standards.

### (iii) Metabolomics data analysis.

The raw data from the machine were assessed using the Xcalibur 4.3 (Thermo Fisher Scientific) Quan Browser software package as done previously (8, 26). A semiquantitative analysis was performed using peak heights of precursor ions with a signal/noise (S/N) ratio of more than 3, a retention time window of less than 60 s, and less than 5 ppm mass error. Height ratios of metabolites were obtained after normalizing the peak heights of the samples to the peak heights of the internal standards used. The absolute concentrations of metabolites were determined using ^12^C-labeled chemical standards (mix of 40 metabolites) as described previously (8, 26). Metabolite concentrations were imputed for missing values and biomass normalized. These values were g-log transformed for identification of the statistically significant metabolites using MetaboAnalyst. Metabolites with an FDR of <0.05 (2-tailed unpaired Student’s *t* test) were considered for further analysis. The absolute metabolite concentrations were expressed as micromoles per gram of DCW or height ratio per gram of DCW.

### NCA.

NCA was performed using the MATLAB scripts and codes made available at GitHub from reference 15 with minor modifications. Only the network component analysis and bootstrap code files were used in this study. NCA predicts TF activity by least-square optimization between the log_10_-transformed gene expression data (in transcripts per million) and the product of connectivity (*a priori*-known interactions between the TF and cognate genes) and TF activity (29). NCA was performed on a smaller data set, especially on the growth-rate specific or indirect gene sets that belonged to the KPE upregulated and downregulated DEGs. As we used a smaller data set, only regulators which were able to reproduce the inferred activity profiles similar to manual interpretation were retained for the analysis. For instance, if a TF positively regulated 4 to 5 genes and is primarily an activator based on the *a priori* knowledge and if this TF showed inferred activity values that indicate a repressor or were inconclusive, then the TF was excluded from further analyses. We were able to successfully reproduce the TF interactions in more than 80% of the TFs across all the growth-rate specific genes in the up- and downregulated KPE DEGs for all the strains. An RNA polymerase that connected all the genes within a set was retained. Analysis of sigma factors such as sigma-70 or sigma-38 along with ppGpp was excluded. The simulation was performed in triplicate for each condition along with bootstrapping to ensure confidence in smaller data sets (70) as well as to keep the data symmetric for correlation with metabolite concentrations. The bootstrap was performed on each TF with 95% confidence intervals across all randomized iterations in each replicate and condition.

### Metabolite-TF interactions.

We analyzed the target enrichments of iTFs available from RegulonDB and EcoCyc in the KPE DEGs restricted to indirect gene targets. The activity of transcriptional regulators was predicted based on the mode of interaction with its target genes. For instance, if the TF is a repressor and the gene targets of the regulator showed a reduction in gene expression, then we inferred that the activity of the TF had increased. A similar analogy was applied in the case of the activator. However, if the TF had less than 70% of its targets positively or negatively regulated, then the regulator was assigned to be a dual regulator. TFs whose interaction with their complete gene lists was not conclusive (<70%) were excluded from the correlation analysis. For instance, if a TF regulated 10 genes, with expression of 7 of them being repressed and expression of 3 being activated, then the TF is regarded as a repressor and retained for analysis. However, if expression of 5 genes was activated and that of the other 5 was repressed, then the TF supposedly functions as a dual regulator, and such regulators were excluded from the correlation analysis. Further, the TFs whose activities were not reproducible in NCA in terms of their manual interpretation were also excluded from the correlation analysis. Next, the metabolites that fell under the direct targets of the focal TFs were retained for correlation analysis. The logic is that the deletion of TF results in changes in gene expression of the direct targets followed by metabolite changes associated with these genes, and these metabolites allosterically affect the inferred activity of other iTFs that regulate part of the growth rate-mediated or indirect gene sets. To identify the direct target-specific metabolites, we performed joint pathway analysis (Metaboanalyst [71]) using both the upregulated and downregulated direct target genes with statistically significant metabolite changes for each of the mutants. Pairs (at least 1 metabolite with many genes or 1 gene with many metabolites) which had an impact value of >0.1 (Metaboanalyst) were retained for the correlation analysis (cutoff arbitrarily set). A Pearson correlation analysis was performed to identify the metabolite-TF interactions. This correlation analysis represents the possibility of occurrence of metabolite-TF interaction and require experimental confirmation. An initial screening of metabolite-TF interactions involved selecting metabolite-TF pairs that had a Pearson correlation coefficient of >0.75 but were statistically significant (*P* < 0.05). Later, those that had a correlation coefficient [*r*] of >0.9 and were statistically significant (*P* < 0.01) and those obtained from literature but found to be significant (*r* > 0.8, *P* < 0.05) in our data set were retained for further analysis.

### WGCNA analysis on compendium gene expression data.

The compendium gene expression data for E. coli K-12 were obtained from the COLOMBOS database (20). COLOMBOS has gene expression data from microarray and transcriptome sequencing (RNA-seq) experiments for various environmental conditions and is further normalized to make it comparable across these conditions. To make the data suitable for coexpression network analysis, the data without centering or scaling were used to impute missing values for the large data set using the global SVD impute function (72, 73). This was performed using pcaMethods (74) with the following parameters: no. of principle components = 5, threshold = 0.01, maxSteps = 1,000, cross-validation cv = “q2”. Further, the expression data were assessed by the goodSamplegenes function from the WGCNA (31) package in R. Finally, the total numbers of genes and samples considered for each organism were 4,189 genes and 4,077 samples. For construction of coexpression networks, the scale-free topology was computed using the pickSoftThreshold function and biweight midcorrelation with default parameters to obtain robust modules from a larger data set. This was followed by construction of a signed topology overlap matrix (TOM). The coexpressed genes were clustered into modules using the flashClust function with the minimum module size set to 30. The cuttreeDynamic function was used to merge the highly similar modules based on eigengenes and a correlation value of 0.75. Each module was represented using a color, and if not mentioned otherwise, the color gray corresponds to uncorrelated genes. In the figures and for all downstream analysis, the genes that belong to a module color and are regulated by iTF (whether enriched or not) are shown/categorized separately. For coexpression analysis, the module colors which had at least 5 genes were considered. Gene ontology analysis was performed on the gene list assigned to a module color considering the above-mentioned criteria.

### Regulator prediction for promoters of direct gene targets.

The potential direct targets which were obtained from chemostat analysis and which did not have reported literature (EcoCyc) or experimental backing were reanalyzed using the FIMO tool (5.4.1) from the MEME suite. The consensus motif used for the analysis for FNR (5′-TTGATNNNNATCAA-3′ or 5′-TTGATYWNNATCAA-3′), ArcA (5′-GTTAATTAAATGTTA-3′ or 5′-GTTAAWWAAATGTTA-3′), and IHF (5′-DTWYYYNGYNNATTTW-3′ or 5′-WWWNWWNWTNTTW-3′) regulators was obtained from the literature (22–25, 27, 44, 45). Statistically significant motifs (*P* < 0.01) were retained such that the binding sites were strictly within 250 bp upstream of the transcription start site of the target gene. Any motif found within the coding region of any gene was excluded from the analysis.

### Sigma factor prediction for promoters of indirect gene targets.

Indirect genes for which no specific global or local regulator could be assigned by either database or for which, despite the presence of binding sites, the regulators were not significantly enriched were considered to be just under the control of RNA polymerase sigma factors. Promoter prediction was done either using iPromoter2L (54, 55) for genes that did not have an annotated RNA polymerase sigma binding site or predictions from EcoCyc. An upstream region 200 bp from the start codon of the gene was chosen for the analysis. The region size (200 to 81 bp) was accordingly adjusted such that the regions did not overlap any coding or promoter regions of other genes. If there was no annotation of any of the sigma factors or if genes were arranged in an operon, then the region upstream of the first gene or the nearest gene was used for the sigma factor identification. The choice and order of arrangement of the predicted sigma factors were done based on the frequency and closeness to the transcriptional start site.

### Data availability.

The RNA sequencing data and the processed files from this study are available at NCBI Geo (https://www.ncbi.nlm.nih.gov/geo/query/acc.cgi?acc) with accession number GSE195954. The metabolomics data presented in this study are available at the NIH Common Fund’s National Metabolomics Data Repository (NMDR) website, the Metabolomics Workbench (https://www.metabolomicsworkbench.org), where it has been assigned project ID PR001316.

## References

[1] Seshasayee ASN, Sivaraman K, Luscombe NM. 2011. An overview of prokaryotic transcription factors: a summary of function and occurrence in bacterial genomes. Subcell Biochem 52:7–23. doi:10.1007/978-90-481-9069-0_2. 21557077

[2] Babu MM, Luscombe NM, Aravind L, Gerstein M, Teichmann SA. 2004. Structure and evolution of transcriptional regulatory networks. Curr Opin Struct Biol 14:283–291. doi:10.1016/j.sbi.2004.05.004. 15193307

[3] Martínez-Antonio A, Collado-Vides J. 2003. Identifying global regulators in transcriptional regulatory networks in bacteria. Curr Opin Microbiol 6:482–489. doi:10.1016/j.mib.2003.09.002. 14572541

[4] Grainger DC, Hurd D, Harrison M, Holdstock J, Busby SJW. 2005. Studies of the distribution of Escherichia coli cAMP-receptor protein and RNA polymerase along the E. coli chromosome. Proc Natl Acad Sci USA 102:17693–17698. doi:10.1073/pnas.0506687102. 16301522 PMC1308901

[5] Utrilla J, O’Brien EJ, Chen K, McCloskey D, Cheung J, Wang H, Armenta-Medina D, Feist AM, Palsson BO. 2016. Global rebalancing of cellular resources by pleiotropic point mutations illustrates a multi-scale mechanism of adaptive evolution. Cell Syst 2:260–271. doi:10.1016/j.cels.2016.04.003. 27135538 PMC4853925

[6] Berthoumieux S, De Jong H, Baptist G, Pinel C, Ranquet C, Ropers D, Geiselmann J. 2013. Shared control of gene expression in bacteria by transcription factors and global physiology of the cell. Mol Syst Biol 9:634. doi:10.1038/msb.2012.70. 23340840 PMC3564261

[7] Gerosa L, Kochanowski K, Heinemann M, Sauer U. 2013. Dissecting specific and global transcriptional regulation of bacterial gene expression. Mol Syst Biol 9:658. doi:10.1038/msb.2013.14. 23591774 PMC3658269

[8] Pal A, Iyer MS, Srinivasan S, Seshasayee ASN, Venkatesh KV. 2022. Global pleiotropic effects in adaptively evolved Escherichia coli lacking CRP reveal molecular mechanisms that define the growth physiology. Open Biol 12:210206. doi:10.1098/rsob.210206. 35167766 PMC8846999

[9] Li Z, Pan Q, Xiao Y, Fang X, Shi R, Fu C, Danchin A, You C. 2019. Deciphering global gene expression and regulation strategy in Escherichia coli during carbon limitation. Microb Biotechnol 12:360–376. doi:10.1111/1751-7915.13343. 30536863 PMC6390033

[10] Pan Q, Li Z, Ju X, Hou C, Xiao Y, Shi R, Fu C, Danchin A, You C. 2021. Escherichia coli segments its controls on carbon-dependent gene expression into global and specific regulations. Microb Biotechnol 14:1084–1106. doi:10.1111/1751-7915.13776. 33650807 PMC8085971

[11] Klumpp S, Zhang Z, Hwa T. 2009. Growth rate-dependent global effects on gene expression in bacteria. Cell 139:1366–1375. doi:10.1016/j.cell.2009.12.001. 20064380 PMC2818994

[12] Yu R, Vorontsov E, Sihlbom C, Nielsen J. 2021. Quantifying absolute gene expression profiles reveals distinct regulation of central carbon metabolism genes in yeast. Elife 10:e65722. doi:10.7554/eLife.65722. 33720010 PMC8016476

[13] Martínez-Antonio A. 2011. Escherichia coli transcriptional regulatory network. Netw Biol 1:21–33. 10.1016/j.jmb.2008.05.054 PMC2726282 18599074

[14] Link H, Kochanowski K, Sauer U. 2013. Systematic identification of allosteric protein-metabolite interactions that control enzyme activity in vivo. Nat Biotechnol 31:357–361. doi:10.1038/nbt.2489. 23455438

[15] Lempp M, Farke N, Kuntz M, Freibert SA, Lill R, Link H. 2019. Systematic identification of metabolites controlling gene expression in E. coli. Nat Commun 10:4463. doi:10.1038/s41467-019-12474-1. 31578326 PMC6775132

[16] Martin BJ, Wolfram L, Matthieu J, Markus U, Jan M, Eric B, Bernd H, Jacobus KR, Ludovic LC, François L, Ulrike M, Pierre N, Sjouke P, Frank R, Dörte B, Philippe B, Elena B, DE L, Etienne DMDK, Geoff D, Samuel D, Liza F, Fm J, Anne G, Annette H, Hc R, Michael H, Sebastian H, Claus H, Hanne J, Edda K, Aurélie L, Peter L, Frank M, Philippe N, Sabine P, Nathalie P, Susanne P, Simon R, Bernd R, Marc S, Julian S, Benno S, Maarten VDJ, Patrick V, Sean WJWA, Jörg S, Stéphane A, et al. 2012. Global network reorganization during dynamic adaptations of Bacillus subtilis metabolism. Science 335:1099–1103. doi:10.1126/science.1206871. 22383848

[17] Chubukov V, Gerosa L, Kochanowski K, Sauer U. 2014. Coordination of microbial metabolism. Nat Rev Microbiol 12:327–340. doi:10.1038/nrmicro3238. 24658329

[18] Kochanowski K, Gerosa L, Brunner SF, Christodoulou D, Nikolaev YV, Sauer U. 2017. Few regulatory metabolites coordinate expression of central metabolic genes in Escherichia coli. Mol Syst Biol 13:903. doi:10.15252/msb.20167402. 28049137 PMC5293157

[19] Tsai MJ, Wang JR, Yang CD, Kao KC, Huang WL, Huang HY, Tseng CP, Huang HD, Ho SY. 2018. PredCRP: predicting and analysing the regulatory roles of CRP from its binding sites in Escherichia coli. Sci Rep 8:951. doi:10.1038/s41598-017-18648-5. 29343727 PMC5772556

[20] Meysman P, Sonego P, Bianco L, Fu Q, Ledezma-Tejeida D, Gama-Castro S, Liebens V, Michiels J, Laukens K, Marchal K, Collado-Vides J, Engelen K. 2014. COLOMBOS v2.0: an ever expanding collection of bacterial expression compendia. Nucleic Acids Res 42:649–653. doi:10.1093/nar/gkt1086. PMC3965013 24214998

[21] Kang Y, Weber KD, Qiu Y, Kiley PJ, Blattner FR. 2005. Genome-wide expression analysis indicates that FNR of Escherichia coli K-12 regulates a large number of genes of unknown function. J Bacteriol 187:1135–1160. doi:10.1128/JB.187.3.1135-1160.2005. 15659690 PMC545700

[22] Myers KS, Yan H, Ong IM, Chung D, Liang K, Tran F, Keleş S, Landick R, Kiley PJ. 2013. Genome-scale analysis of Escherichia coli FNR reveals complex features of transcription factor binding. PLoS Genet 9:11–13. doi:10.1371/journal.pgen.1003565. PMC3688515 23818864

[23] Park DM, Akhtar MS, Ansari AZ, Landick R, Kiley PJ. 2013. The bacterial response regulator ArcA uses a diverse binding site architecture to regulate carbon oxidation globally. PLoS Genet 9:e1003839. doi:10.1371/journal.pgen.1003839. 24146625 PMC3798270

[24] Federowicz S, Kim D, Ebrahim A, Lerman J, Nagarajan H, Cho BK, Zengler K, Palsson B. 2014. Determining the control circuitry of redox metabolism at the genome-scale. PLoS Genet 10:e1004264. doi:10.1371/journal.pgen.1004264. 24699140 PMC3974632

[25] Grainger DC, Aiba H, Hurd D, Browning DF, Busby SJW. 2007. Transcription factor distribution in Escherichia coli: studies with FNR protein. Nucleic Acids Res 35:269–278. doi:10.1093/nar/gkl1023. 17164287 PMC1802558

[26] Iyer MS, Pal A, Srinivasan S, Somvanshi PR. 2021. Global transcriptional regulators fine-tune the translational and metabolic efficiency for optimal growth of Escherichia coli. mSystems 6:e00001-21. doi:10.1128/mSystems.00001-21. 33785570 PMC8546960

[27] Prieto AI, Kahramanoglou C, Ali RM, Fraser GM, Seshasayee ASN, Luscombe NM. 2012. Genomic analysis of DNA binding and gene regulation by homologous nucleoid-associated proteins IHF and HU in Escherichia coli K12. Nucleic Acids Res 40:3524–3537. doi:10.1093/nar/gkr1236. 22180530 PMC3333857

[28] Kahramanoglou C, Seshasayee ASN, Prieto AI, Ibberson D, Schmidt S, Zimmermann J, Benes V, Fraser GM, Luscombe NM. 2011. Direct and indirect effects of H-NS and Fis on global gene expression control in Escherichia coli. Nucleic Acids Res 39:2073–2091. doi:10.1093/nar/gkq934. 21097887 PMC3064808

[29] Liao JC, Boscolo R, Yang YL, Tran LM, Sabatti C, Roychowdhury VP. 2003. Network component analysis: reconstruction of regulatory signals in biological systems. Proc Natl Acad Sci USA 100:15522–15527. doi:10.1073/pnas.2136632100. 14673099 PMC307600

[30] Liu W, Li L, Long X, You W, Zhong Y, Wang M, Tao H, Lin S, He H. 2018. Construction and analysis of gene co-expression networks in Escherichia coli. Cells 7:19. doi:10.3390/cells7030019. 29518040 PMC5870351

[31] Langfelder P, Horvath S. 2008. WGCNA: an R package for weighted correlation network analysis. BMC Bioinformatics 9:559. doi:10.1186/1471-2105-9-559. 19114008 PMC2631488

[32] Galán-Vásquez E, Perez-Rueda E. 2019. Identification of modules with similar gene regulation and metabolic functions based on co-expression data. Front Mol Biosci 6:139. doi:10.3389/fmolb.2019.00139. 31921888 PMC6929668

[33] Datsenko KA, Wanner BL. 2000. One-step inactivation of chromosomal genes in Escherichia coli K-12 using PCR products. Proc Natl Acad Sci USA 97:6640–6645. doi:10.1073/pnas.120163297. 10829079 PMC18686

[34] Covert MW, Knight EM, Reed JL, Herrgard MJ, Palsson BO. 2004. Integrating high-throughput and computational data elucidates bacterial networks. Nature 429:92–96. doi:10.1038/nature02456. 15129285

[35] Liebermeister W, Noor E, Flamholz A, Davidi D, Bernhardt J, Milo R. 2014. Visual account of protein investment in cellular functions. Proc Natl Acad Sci USA 111:8488–8493. doi:10.1073/pnas.1314810111. 24889604 PMC4060655

[36] Tweeddale H, Notley-Mcrobb L, Ferenci T. 1998. Effect of slow growth on metabolism of Escherichia coli, as revealed by global metabolite pool (‘metabolome’) analysis. J Bacteriol 180:5109–5116. doi:10.1128/JB.180.19.5109-5116.1998. 9748443 PMC107546

[37] Metzl-Raz E, Kafri M, Yaakov G, Soifer I, Gurvich Y, Barkai N. 2017. Principles of cellular resource allocation revealed by condition-dependent proteome profiling. eLife 6:e28034. doi:10.7554/eLife.28034. 28857745 PMC5578734

[38] Scott M, Klumpp S, Mateescu EM, Hwa T. 2014. Emergence of robust growth laws from optimal regulation of ribosome synthesis. Mol Syst Biol 10:747. doi:10.15252/msb.20145379. 25149558 PMC4299513

[39] Dai X, Zhu M, Warren M, Balakrishnan R, Okano H, Williamson JR, Fredrick K, Hwa T. 2018. Slowdown of translational elongation in Escherichia coli under hyperosmotic stress. mBio 9:e02375-17. doi:10.1128/mBio.02375-17. 29440576 PMC5821080

[40] Dai X, Zhu M, Warren M, Balakrishnan R, Patsalo V, Okano H, Williamson JR, Fredrick K, Wang Y-P, Hwa T. 2016. Reduction of translating ribosomes enables Escherichia coli to maintain elongation rates during slow growth. Nat Microbiol 2:16231. doi:10.1038/nmicrobiol.2016.231. 27941827 PMC5346290

[41] Li SH, Li Z, Park JO, King CG, Rabinowitz JD, Wingreen NS, Gitai Z. 2018. Escherichia coli translation strategies differ across carbon, nitrogen and phosphorus limitation conditions. Nat Microbiol 3:939–947. doi:10.1038/s41564-018-0199-2. 30038306 PMC6278830

[42] Zhang Q, Brambilla E, Shi H, Lagomarsino MC, Sclavi B. 2020. A decrease in transcription capacity limits growth rate upon translation inhibition. mSystems 5:e00575-20. doi:10.1128/mSystems.00575-20. 32900870 PMC7483510

[43] Grant CE, Bailey TL, Noble WS. 2011. FIMO: scanning for occurrences of a given motif. Bioinformatics 27:1017–1018. doi:10.1093/bioinformatics/btr064. 21330290 PMC3065696

[44] Keseler IM, Mackie A, Santos-Zavaleta A, Billington R, Bonavides-Martínez C, Caspi R, Fulcher C, Gama-Castro S, Kothari A, Krummenacker M, Latendresse M, Muñiz-Rascado L, Ong Q, Paley S, Peralta-Gil M, Subhraveti P, Velázquez-Ramírez DA, Weaver D, Collado-Vides J, Paulsen I, Karp PD. 2017. The EcoCyc database: reflecting new knowledge about Escherichia coli K-12. Nucleic Acids Res 45:D543–D550. doi:10.1093/nar/gkw1003. 27899573 PMC5210515

[45] Santos-Zavaleta A, Salgado H, Gama-Castro S, Sánchez-Pérez M, Gómez-Romero L, Ledezma-Tejeida D, García-Sotelo JS, Alquicira-Hernández K, Muñiz-Rascado LJ, Peña-Loredo P, Ishida-Gutiérrez C, Velázquez-Ramírez DA, Del Moral-Chávez V, Bonavides-Martínez C, Méndez-Cruz CF, Galagan J, Collado-Vides J. 2019. RegulonDB v 10.5: tackling challenges to unify classic and high throughput knowledge of gene regulation in E. coli K-12. Nucleic Acids Res 47:D212–D220. doi:10.1093/nar/gky1077. 30395280 PMC6324031

[46] Choudhary KS, Kleinmanns JA, Decker K, Sastry AV, Gao Y, Szubin R, Seif Y, Palsson BO. 2020. Elucidation of regulatory modes for five two-component systems in Escherichia coli reveals novel relationships. mSystems 5:e00980-20. doi:10.1128/mSystems.00980-20. 33172971 PMC7657598

[47] Schmidt A, Kochanowski K, Vedelaar S, Ahrné E, Volkmer B, Callipo L, Knoops K, Bauer M, Aebersold R, Heinemann M. 2016. The quantitative and condition-dependent Escherichia coli proteome. Nat Biotechnol 34:104–110. doi:10.1038/nbt.3418. 26641532 PMC4888949

[48] Yu R, Campbell K, Pereira R, Björkeroth J, Qi Q, Vorontsov E, Sihlbom C, Nielsen J. 2020. Nitrogen limitation reveals large reserves in metabolic and translational capacities of yeast. Nat Commun 11:1–12. doi:10.1038/s41467-020-15749-0. 32312967 PMC7171132

[49] Reznik E, Christodoulou D, Goldford JE, Briars E, Sauer U, Segrè D, Noor E. 2017. Genome-scale architecture of small molecule regulatory networks and the fundamental trade-off between regulation and enzymatic activity. Cell Rep 20:2666–2677. doi:10.1016/j.celrep.2017.08.066. 28903046 PMC5600504

[50] Zampieri M, Hörl M, Hotz F, Müller NF, Sauer U. 2019. Regulatory mechanisms underlying coordination of amino acid and glucose catabolism in Escherichia coli. Nat Commun 10:3354. doi:10.1038/s41467-019-11331-5. 31350417 PMC6659692

[51] McCloskey D, Xu S, Sandberg TE, Brunk E, Hefner Y, Szubin R, Feist AM, Palsson BO. 2018. Adaptive laboratory evolution resolves energy depletion to maintain high aromatic metabolite phenotypes in Escherichia coli strains lacking the phosphotransferase system. Metab Eng 48:233–242. doi:10.1016/j.ymben.2018.06.005. 29906504

[52] Deutscher J, Francke C, Postma PW, Francke C, Postma PW. 2006. How phosphotransferase system-related protein phosphorylation regulates carbohydrate metabolism in bacteria. Microbiol Mol Biol Rev 70:939–1031. doi:10.1128/MMBR.00024-06. 17158705 PMC1698508

[53] Postma PW, Lengeler JW, Jacobson GR. 1993. Phosphoenolpyruvate:carbohydrate phosphotransferase systems of bacteria. Microbiol Rev 57:543–594. doi:10.1128/mr.57.3.543-594.1993. 8246840 PMC372926

[54] Liu B, Yang F, Huang D-S, Chou K-C. 2018. iPromoter-2L: a two-layer predictor for identifying promoters and their types by multi-window-based PseKNC. Bioinformatics 34:33–40. doi:10.1093/bioinformatics/btx579. 28968797

[55] Chen W, Lei T-Y, Jin D-C, Lin H, Chou K-C. 2014. PseKNC: a flexible web server for generating pseudo K-tuple nucleotide composition. Anal Biochem 456:53–60. doi:10.1016/j.ab.2014.04.001. 24732113

[56] Imam S, Noguera DR, Donohue TJ. 2015. An integrated approach to reconstructing genome-scale transcriptional regulatory networks. PLoS Comput Biol 11:e1004103. doi:10.1371/journal.pcbi.1004103. 25723545 PMC4344238

[57] Hua Q, Yang C, Oshima T, Mori H, Shimizu K. 2004. Analysis of gene expression in Escherichia coli in response to changes of growth-limiting nutrient in chemostat cultures. Appl Environ Microbiol 70:2354–2366. doi:10.1128/AEM.70.4.2354-2366.2004. 15066832 PMC383082

[58] Simen JD, Michael L, Takors R, Matthes J, Jan M. 2017. Transcriptional response of Escherichia coli to ammonia and glucose fluctuations. Microb Biotechnol 10:858–872. doi:10.1111/1751-7915.12713. 28447391 PMC5481515

[59] Diether M, Nikolaev YV, Allain HF, Sauer U. 2019. Systematic mapping of protein-metabolite interactions in central metabolism of Escherichia coli. Mol Syst Biol 15:e9008. 31464375 10.15252/msb.20199008 PMC6706640

[60] Wang J, Rennie W, Liu C, Carmack CS, Prévost K, Caron MP, Massé E, Ding Y, Wade JT. 2015. Identification of bacterial sRNA regulatory targets using ribosome profiling. Nucleic Acids Res 43:10308–10320. doi:10.1093/nar/gkv1158. 26546513 PMC4666370

[61] Arifuzzaman M, Maeda M, Itoh A, Nishikata K, Takita C, Saito R, Ara T, Nakahigashi K, Huang H, Hirai A, Tsuzuki K, Nakamura S, Altaf-Ul-Amin M, Oshima T, Baba T, Yamamoto N, Kawamura T, Ioka-Nakamichi T, Kitagawa M, Tomita M, Kanaya S, Wada C, Mori H. 2006. Large-scale identification of protein–protein interaction of Escherichia coli K-12. Genome Res 16:686–691. doi:10.1101/gr.4527806. 16606699 PMC1457052

[62] Taylor TB, Shepherd MJ, Jackson RW, Silby MW. 2022. Natural selection on crosstalk between gene regulatory networks facilitates bacterial adaptation to novel environments. Curr Opin Microbiol 67:102140. doi:10.1016/j.mib.2022.02.002. 35248980

[63] Martin M. 2011. Cutadapt removes adapter sequences from high-throughput sequencing reads. EMBnet J 17:10–12. doi:10.14806/ej.17.1.200.

[64] Li H, Durbin R. 2009. Fast and accurate short read alignment with Burrows-Wheeler transform. Bioinformatics 25:1754–1760. doi:10.1093/bioinformatics/btp324. 19451168 PMC2705234

[65] Li H, Handsaker B, Wysoker A, Fennell T, Ruan J, Homer N, Marth G, Abecasis G, Durbin R, 1000 Genome Project Data Processing Subgroup. 2009. The Sequence Alignment/Map format and SAMtools. Bioinformatics 25:2078–2079. doi:10.1093/bioinformatics/btp352. 19505943 PMC2723002

[66] Liao Y, Smyth GK, Shi W. 2014. FeatureCounts: an efficient general purpose program for assigning sequence reads to genomic features. Bioinformatics 30:923–930. doi:10.1093/bioinformatics/btt656. 24227677

[67] Robinson MD, McCarthy DJ, Smyth GK. 2010. edgeR: a Bioconductor package for differential expression analysis of digital gene expression data. Bioinformatics 26:139–140. doi:10.1093/bioinformatics/btp616. 19910308 PMC2796818

[68] Carbon S, Ireland A, Mungall CJ, Shu S, Marshall B, Lewis S, Lomax J, Mungall C, Hitz B, Balakrishnan R, Dolan M, Wood V, Hong E, Gaudet P, Web Presence Working Group. 2009. AmiGO: online access to ontology and annotation data. Bioinformatics 25:288–289. doi:10.1093/bioinformatics/btn615. 19033274 PMC2639003

[69] Alexa A, Rahnenfuhrer J. 2020. topGO: enrichment analysis for Gene Ontology doi:10.18129/B9.bioc.topGO.

[70] Misra A, Sriram G. 2013. Network component analysis provides quantitative insights on an Arabidopsis transcription factor-gene regulatory network. BMC Syst Biol 7:126. doi:10.1186/1752-0509-7-126. 24228871 PMC3843564

[71] Xia J, Psychogios N, Young N, Wishart DS. 2009. MetaboAnalyst: a web server for metabolomic data analysis and interpretation. Nucleic Acids Res 37:W652–W660. doi:10.1093/nar/gkp356. 19429898 PMC2703878

[72] Liew AWC, Law NF, Yan H. 2011. Missing value imputation for gene expression data: computational techniques to recover missing data from available information. Brief Bioinform 12:498–513. doi:10.1093/bib/bbq080. 21156727

[73] Troyanskaya O, Cantor M, Sherlock G, Brown P, Hastie T, Tibshirani R, Botstein D, Altman RB. 2001. Missing value estimation methods for DNA microarrays. Bioinformatics 17:520–525. doi:10.1093/bioinformatics/17.6.520. 11395428

[74] Stacklies W, Redestig H, Scholz M, Walther D, Selbig J. 2007. pcaMethods—a Bioconductor package providing PCA methods for incomplete data. Bioinformatics 23:1164–1167. doi:10.1093/bioinformatics/btm069. 17344241

